# The longitudinal association between working from home and musculoskeletal pain during the COVID-19 pandemic

**DOI:** 10.1007/s00420-022-01946-5

**Published:** 2022-12-25

**Authors:** Esmee Bosma, Bette Loef, Sandra H. van Oostrom, Lifelines Corona Research Initiative, Karin I. Proper

**Affiliations:** 1 Center for Nutrition, Prevention and Health Services, National Institute for Public Health and the Environment, Bilthoven, The Netherlands; 2 Department of Public and Occupational Health, Amsterdam UMC, Vrije Universiteit Amsterdam, Amsterdam Public Health Research Institute, Amsterdam, The Netherlands

**Keywords:** Working from home, Musculoskeletal pain, COVID-19 pandemic, Longitudinal data

## Abstract

**Objective:**

This study investigates the associations between working from home and the presence of MSP during the COVID-19 pandemic. Working from home often involves a lot of sedentary computer screen work and the home working environment might not be optimally equipped, which can lead to health problems, including musculoskeletal pain (MSP).

**Methods:**

Longitudinal data from 16 questionnaire rounds of the Lifelines COVID-19 cohort during the first year of the COVID-19 pandemic (March 2020-February 2021) were used. In total, 40,702 Dutch workers were included. In every round, participants reported whether they worked on location, from home, or hybrid. Logistic Generalized Estimating Equations were used to study the association of work situation with the presence of MSP and the presence of severe MSP.

**Results:**

Working from home was associated with higher risks of having MSP in the lower back (OR: 1.05, 95% CI 1.02–1.08), in the upper back (OR: 1.24, 95% CI 1.18–1.31), and in the neck, shoulder(s) and/or arm(s) (OR: 1.18, 95% CI 1.13–1.22). Hybrid working was associated with higher risks of having pain in the upper back (OR: 1.09, 95% CI 1.02–1.17) and in the neck, shoulder(s) and/or arm(s) (OR: 1.14, 95% CI 1.09–1.20). Both home and hybrid workers had higher risks of severe MSP in the different body areas.

**Conclusion:**

Home workers, and to a smaller extent hybrid workers, had higher risks of having MSP than location workers during the first year of the COVID-19 pandemic. The results indicate the importance of measures to prevent MSP in future policies involving working from home.

## Introduction

During the COVID-19 pandemic, different containment measures were implemented to control the spread of the SARS-CoV-2 virus, including working from home as much as possible. As a result, working arrangements changed and the number of people that worked from home increased tremendously (Leroy et al. 2021; Lopez-Leon et al. 2020). To illustrate, in The Netherlands before the COVID-19 pandemic in 2019, 37 percent of the workers did work from home sometimes, on average for slightly more than 6 h per week and working completely from home barely happened (Hooftman et al. 2020). By mid-2020, the proportion of people working from home increased to 45 percent with most of them working completely from home, with an average of 29 h per week (Hooftman et al. 2020).

Besides the advantages of working from home (e.g. less commuting and having more flexibility in work hours), working from home brings several constraints (Ipsen et al. 2021). First, working from home often involves a lot of sedentary computer screen work with less active interruptions, for example to walk to meetings or go to the printer, than when working at location. In addition, only one-third of home workers in 2020 and half of home workers in 2021 in the Netherlands had an optimally furnished workplace for a good work posture; an adjustable desk, an adjustable chair, a separate monitor and a separate mouse all together (Hooftman et al. 2020; Oude Hengel et al. 2021). A large group of home workers thus lack an ergonomically sound workplace at home. Furthermore, when ergonomic and adaptable furniture is available, workers do not always install and use the furniture appropriately (van Niekerk et al. 2012). Sitting for many hours behind the screen at workplaces which are not ergonomically installed subsequently creates a risk of musculoskeletal pain (MSP) (van Niekerk et al. 2012; Wahlström 2005). Based on these factors together, one would expect that homeworkers experienced more MSP compared to location workers during the COVID-19 pandemic. 

Not much is known yet about the association between working from home and MSP during the COVID-19 pandemic and recently conducted studies showed mixed results. A Turkish case-controlled study showed that pain in the lower back was higher among home workers in comparison with location workers during 3-month lockdown from March 2020 onwards (Toprak Celenay et al. 2020). Results of other European cross-sectional studies comparing MSP during pre- and peri-lockdown indicated no differences (Argus and Pääsuke 2021) or even a decrease (possibly through increased exercise in the study population) in MSP among home workers (Rodríguez-Nogueira et al. 2020). Descriptive results of a Dutch report also showed a decrease of arm, neck or shoulder complaints among homeworkers from 42 percent before the lockdown in 2019 to 38 percent during the lockdown in 2021 (Oude Hengel et al. 2021). This decrease of arm, neck or shoulder complaints was the same for location workers (from 42 percent in 2019 to 36 percent in 2021). However, an ensemble of in-depth studies using appropriate control groups and control variables to study the association between working from home and MSP is not yet available. 

As a consequence of previous studies using a cross-sectional study design, knowledge is lacking about the presence of MSP over time during the COVID-19 pandemic. For example, it is conceivable that the longer time people work from home in probable sub-optimally equipped workplaces, the more MSP will arise, or that the presence of MSP varies according to different containment measures. To get insight into the association between working from home and the presence of MSP during a longer period, multiple measurements over a longer period are needed. Therefore, the aim of our study was to investigate the longitudinal association between working from home and MSP, with multiple measurements during the first year of the COVID-19 pandemic in a large population of Dutch workers. As working from home, either fully or partly, is expected to remain more common practice, knowledge about the impact on MSP is relevant for future policies involving working from home.

## Data and methods

### Data

Data from the Lifelines COVID-19 cohort were used (Lifelines 2021). The Lifelines COVID-19 cohort is part of the larger Lifelines population cohort which is a multi-disciplinary prospective population-based cohort study examining in an unique three-generation design the health and health-related behaviors of 167,729 persons living in the North of the Netherlands (provinces Groningen, Friesland and Drenthe). It employs a broad range of investigative procedures in assessing the biomedical, socio-demographic, behavioral, physical and psychological factors which contribute to the health and disease of the general population, with a special focus on multi-morbidity and complex genetics. During three questionnaire rounds of assessment (1A 2007–2013, 2A 2014–2017, 3A 2019–2023) and additional questionnaires in-between (1B 2011–2014, 1C 2012–2015, 2B 2016–2019) (Sijtsma et al. 2021), participants answered questions including their demographics (e.g., age, sex, educational level) and work-related factors (occupation, occupational status) (Lifelines 2021). 

Active participants aged 18 years or older in the Lifelines population cohort were invited to participate in the Lifelines COVID-19 cohort. The aim of the Lifelines COVID-19 cohort was to study the possible causes of developing serious symptoms in response to an infection with SARS-CoV-2 and the impact of the pandemic and quarantine on physical and mental health and socio-economic status in the general population. Data collection started at the beginning of the COVID-19 pandemic in The Netherlands, in March 2020, and participants were invited to fill in a digital questionnaire each round. Starting in round 8 (May 23, 2020), participants were invited only if they had completed at least one previous COVID-19 questionnaire in rounds 1–7. Data of the same participants in different study rounds could be linked to each other by a pseudonymized linking variable which was provided by the Lifelines COVID-19 cohort. Measurements consisted of (bi-)weekly questionnaires and from September 2020 onwards of monthly questionnaires with questions about work situation and physical and mental wellbeing. 

**Fig. 1:**
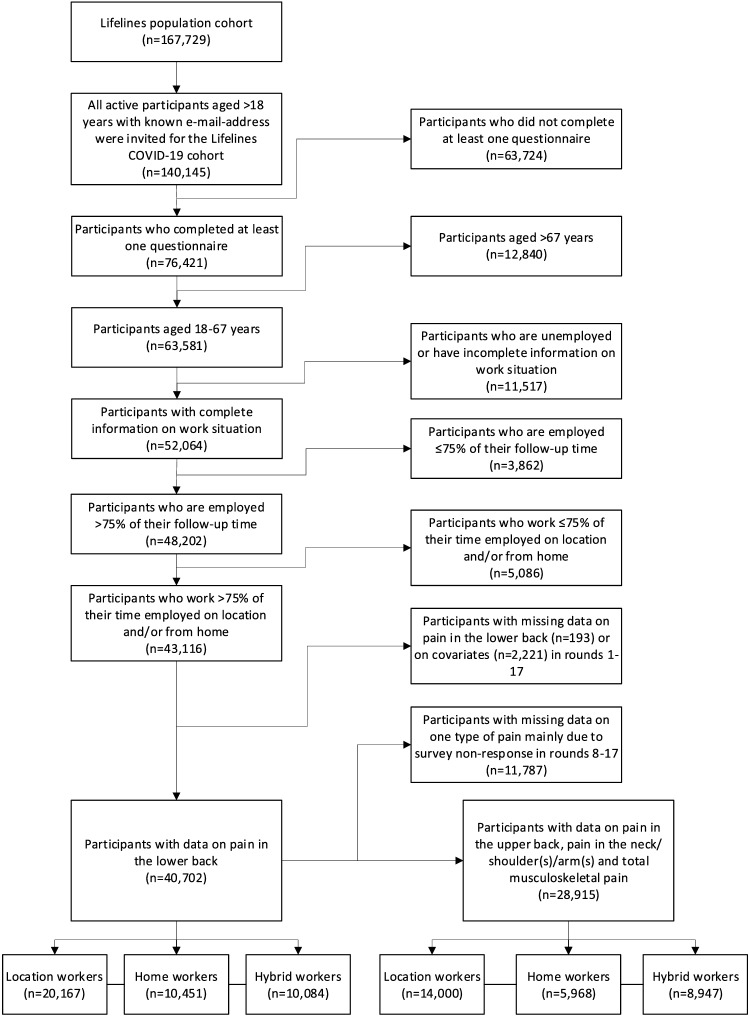
Flow diagram of the study population

In this study, data of 16 questionnaire rounds (conducted between March 2020-February 2021) of the Lifelines COVID-19 cohort were used, for an overview of the used questionnaire rounds, see Appendix A, Table 4. Participants who completed at least one questionnaire and had a working age (between 18 and 67 years) were included (*N* = 63,581). As we aimed to include participants who were active workers for the majority of the study, only workers who worked > 75% of the rounds in which they participated were included (*N* = 48,202). In addition, of the rounds in which they worked, workers needed to work > 75% of the time on location and/or from home (see work situation measure) to be included (*N* = 43,116). Hereafter, participants were included if they had at least one round of data available on the different types of musculoskeletal pain and complete data on the covariates (*N* = 40,702 for lower back pain and *N* = 28,915 for upper back pain and pain in the neck/shoulder(s) and/or arm(s)). See Fig. 1 below for an overview of all selections made.

### Measurements

#### *Work situation*

Work situation was operationalized by a variable with three categories indicating whether a participant was working from home, working on location or working both at home and on location (hybrid) at a specific round. Participants were asked about their daily activity with the following question: ‘What do you currently do in your daily life?’ with the answer categories ‘I am a student’, ‘I work (full-time, part-time, freelance)’, ‘I am on disability’, ‘I am unemployed’, ‘I am retired’, ‘I am on maternity leave’ or ‘other’. Participants who answered their daily activity is work were further asked about their current work situation with multiple answers possible from ‘I work from home’, ‘I am laid off but am still being paid’, ‘I am laid off and am no longer paid’, I continue to work at the usual location (e.g. office, factory, construction site)’, ‘I continue to work at multiple sites for my job’, ‘I am forced to take sick leave or vacation time’ and ‘other’. Participants who answered ‘I work from home’ were categorized as home workers in that specific round. If participants answered ‘I continue to work at the usual location’ or ‘I continue to work at multiple sites for my job’ they were categorized as location workers. When participants answered to work on location as well as from home within one questionnaire round they belonged to the category hybrid workers. This variable was used to visualize the working situation over time and for the GEE analyses.

Next to the ‘round-specific work situation’ variable, an ‘overall work situation’ was created. In this overall work situation variable, participants were categorized as home workers if they worked from home and did not work on location in all the available rounds during the year. Participants were categorized as location workers if they worked on location and did not work from home in all the available rounds. Participants were categorized as hybrid workers if they worked from home as well as on location in the available rounds during the year. The overall work situation variable was used in the descriptive statistics of our population, in the flowchart of selections and in the third sensitivity analysis in which continuous working from home was compared to hybrid working during the overall study period.

#### MSP

MSP was measured by questions that covered different body areas, including the following: lower back, upper back, and pain in the neck, shoulder(s) and/or arm(s). Questions used were derived from the somatization subscale of the Symptom Checklist (SCL-90) (Derogatis et al. 1977; Lifelines 2020). Up to and including the sixth round (March 30, 2020 – May 25, 2020) pain in lower back was assessed by the following question: ‘To what extent have you had the following symptoms in the last 7 days?’ and pain in the lower back followed as one of the symptoms. Respondents could answer on a five-point scale as follows: not at all (0), a little bit (1), somewhat (2), quite a lot (3), or very much (4). From round 7 onwards (May 15, 2020–March 25, 2021) the questionnaire was distributed bi-weekly, so 'the last 7 days' changed to 'the last 14 days’. From round 8 onwards, a question on pain in the upper back and another question on pain in the neck, shoulder(s) and/or arm(s) together were added using a similar question and a five-point answer scale.

Two types of outcome measures were created as follows: presence of pain and presence of severe pain. For the analyses on the presence of pain, the scorings per body area were dichotomized into ‘0 = no pain’ based on the first answer category ‘not at all’ and ‘1 = pain’ consisting of the categories ‘a little bit’ to ‘very much’ combined. In addition, a dichotomous variable ‘total MSP’ was created where 0 means no pain at all in any body area and 1 means the presence of at least a little bit of pain in one or more body areas. For the statistical analyses on severe pain, the scorings per body area if pain occurs were dichotomized into ‘0 = no severe pain’ based on the answer categories ‘not at all’ to ‘somewhat’ and ‘1 = severe pain’ consisting of the categories ‘quite a lot’ and ‘very much’.

#### Covariates

Several demographic covariates and work-related covariates were included as home workers and location workers are likely to differ with regard to their demographic characteristics and their jobs and corresponding work characteristics, as has been previously shown (Adams-Prassl et al. 2022). In addition, MSP may depend on the type of work and amount of physical strain (Lis et al. 2007). Sex, age (in years), educational attainment (low, middle or high), country of birth (within or outside The Netherlands) and household composition (‘living alone’, ‘living together with adults’, ‘living together with children’, ‘living together with children and adults’ or ‘living together but unknown with whom’) were included as demographic covariates. Occupational class (‘high-skilled white-collar’, ‘low-skilled white-collar’, ‘high-skilled blue collar’ and ‘low-skilled blue-collar’) and type of occupation (Appendix A, Table 5), both based on the International Standard Classification of Occupations (ISCO), and type of contract over the study period (‘exclusively a permanent contract’, ‘both a permanent and non-permanent contract’ and ‘exclusively a non-permanent contract’) were included as work covariates. Last, body mass index (BMI) was included, which may be related to MSP (Aro and Leino 1985; Kortt and Baldry 2002). 

### Statistical analyses

Descriptive analyses of the round-specific work situation were conducted to determine the amount of home working over the study period. Descriptive analyses separately for the three groups of overall work situation were conducted to get insight into the characteristics of our study population. Then, unadjusted graphs of trend lines were created to observe MSP over time for the different pain types, for the frequencies of the presence of MSP, as well as for the severity of MSP when pain occurs. To study the association between working from home and each dichotomized MSP outcome, data were analyzed with logistic generalized estimating equations (GEE) with an exchangeable correlation structure. For each outcome, we used the following three models: a crude model (model 0), a model adjusted for demographic variables (model 1) and a fully adjusted model (model 2) with additional adjustment for work variables and BMI. In these models, we used the round-specific work situation variable with location workers as reference group. 

Finally, three sensitivity analyses were performed. First, to check if the association between working from home and MSP was confounded by workers’ general health, self-perceived general health was included as covariate in a subsample of the population (*N* = 33,411) (Hestbaek et al. 2003). General health was measured with the question ‘How would you rate your health, generally speaking?’ and dichotomized into ‘0 = poor general health’ based on the answer categories ‘poor’ and ‘fair’ and ‘1 = good general health’ consisting of the categories ‘good’, ‘very good’, and ‘excellent’. Home workers (95.2%) and hybrid workers (95.9%) reported to have a good general health somewhat less often than location workers (96.4%) (*p* < 0.05). Second, to test whether the results hold in a group which is relatively more homogeneous considering occupation, we conducted a sensitivity analysis among workers in business economics and administrative occupations (e.g. accountants, policy advisors, administrative officers, *N* = 9557). Third, in order to test whether the risks of MSP and/or severe MSP are higher when someone worked at home during all available rounds of the study period compared to also having worked occasionally on location, an analysis was performed using the overall working situation variable with overall hybrid working as reference group (*N* = 40,702 for lower back pain and *N* = 28,915 for upper back pain and pain in the neck/shoulder(s) and/or arm(s)). The software IBM SPSS 25 was used to conduct the analyses.

### Results

#### Descriptive results of the study population

In the first questionnaire round, 44 percent of the participants worked exclusively from home. The percentage of the participants working from home decreased to 21 percent in September 2020 and increased again to 33 percent at end of the study period. Table 1 shows the characteristics of the workers: 58.7 percent were females, the mean age was 49 years and only a minority had a low educational level (13.0%). The largest differences in demographic variables by work situation were found in educational level (66.4% of home workers and 64.5% of hybrid workers had a high educational level compared to 24.2% of location workers) and occupational class (73.7% of home workers and 72.2% of hybrid workers were employed in high-skilled white-collar occupations compared to 42.7% of location workers).

**Table 1:** Characteristics of the study population by work situation

	Home workers (*N* = 10,456)	Hybrid workers (*N* = 10,084)	Location workers (*N* = 20,167)	Total (*N* = 40,702)
Female (%)	55.6*	60.5	59.4	58.7
Age (mean, SD)	48.36* (9.32)	48.67* (9.49)	50.09 (8.96)	49.29 (9.22)
Educational level (%)				
Low	4.9*	5.0*	21.2	13.0
Middle	28.7*	30.5*	54.6	42.0
High	66.4*	64.5*	24.2	45.0
Country of birth The Netherlands (%)	97.4*	98.2	98.1	98.0
Household composition (%)				
Living alone	7.7*	6.9*	7.8	7.5
Living together with children	2.2*	1.9*	1.3	1.7
Living together with adults	38.2*	43.0*	43.8	42.1
Living together with children and adults	37.1*	39.3*	31.1	34.7
Living together but unknown with whom	14.7*	8.9*	16.0	13.9
Occupation (%)				
High-skilled white-collar	73.7*	72.2*	42.7	57.9
Low-skilled white-collar	22.2*	22.5*	33.5	27.9
High-skilled blue collar	2.5*	3.6*	12.0	7.5
Low-skilled blue-collar	1.6*	1.8*	11.8	6.7
Type of contract (%)				
Permanent	75.5*	72.0*	79.2	76.4
Temporary	4.5*	10.6*	5.7	6.6
Combination	20.0*	17.5*	15.1	17.0
BMI (mean, SD)	25.88* (4.27)	25.86* (4.19)	26.38 (4.28)	26.12 (4.26)

Numeric variables were reported by mean (standard deviation), and frequency (%) was given for categorical variables. Workers are categorized as home workers if they were working from home on each point during the pandemic, as location workers if they were working on location on each point during the pandemic and as hybrid workers when they were both working from home and on working on location at some point during the pandemic

*p<0.05; statistically significant difference between home workers and location workers or between hybrid workers and location workers, tested with independent−samples t−test and chi−square test

### Presence of MSP and presence of severe MSP during the first year of the pandemic

**Fig. 2:**
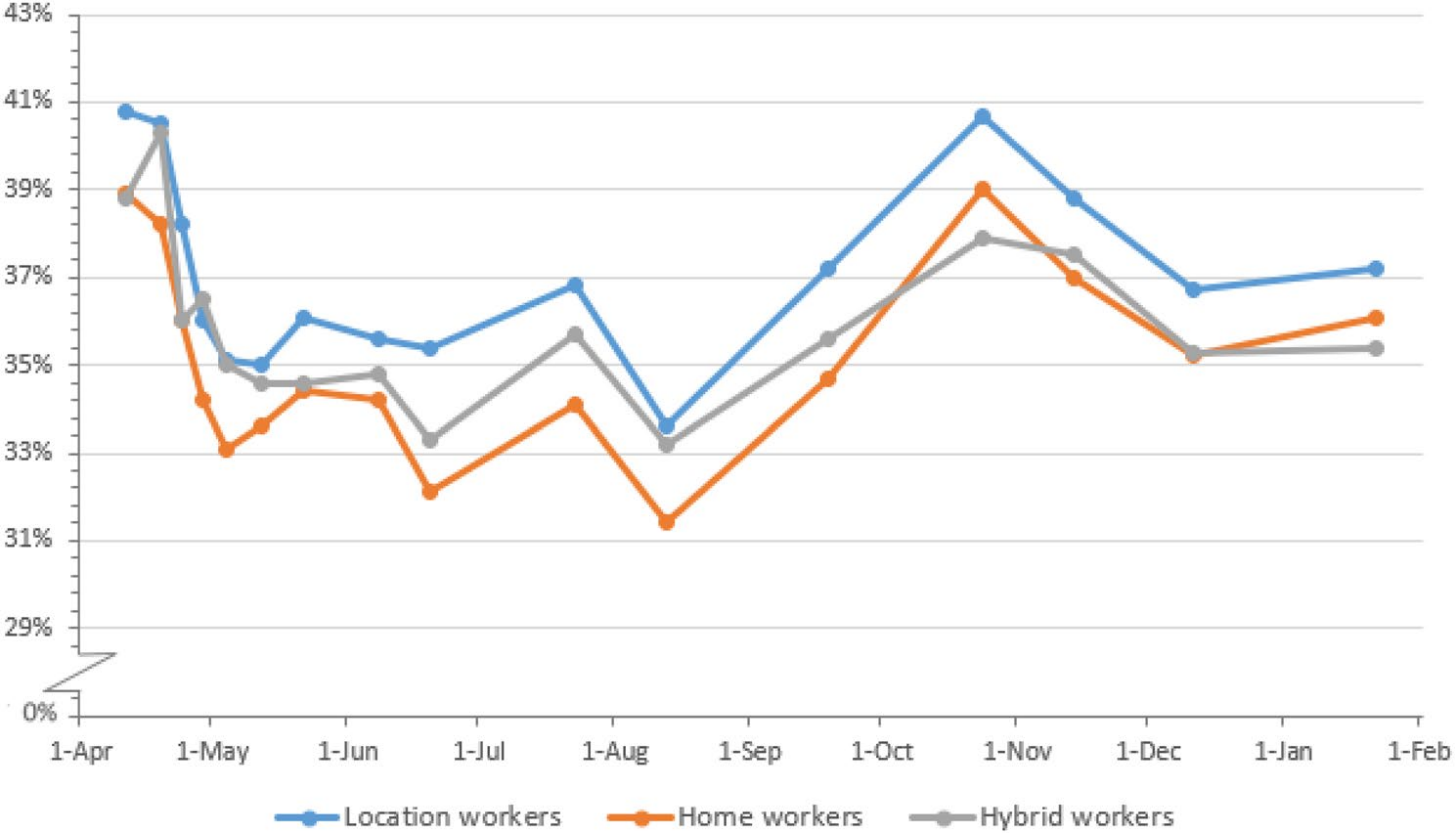
Percentages of participants with pain in the lower back during the pandemic from April 2020 until February 2021, by work situation and date. *N* per round is, respectively, 24,210, 23,811, 23,418, 23,776, 22,286, 20,393, 20,661, 17,244, 16,435, 15,708, 16,564, 16,888, 15,774, 15,807, 14,575, 15,530

**Fig. 3:**
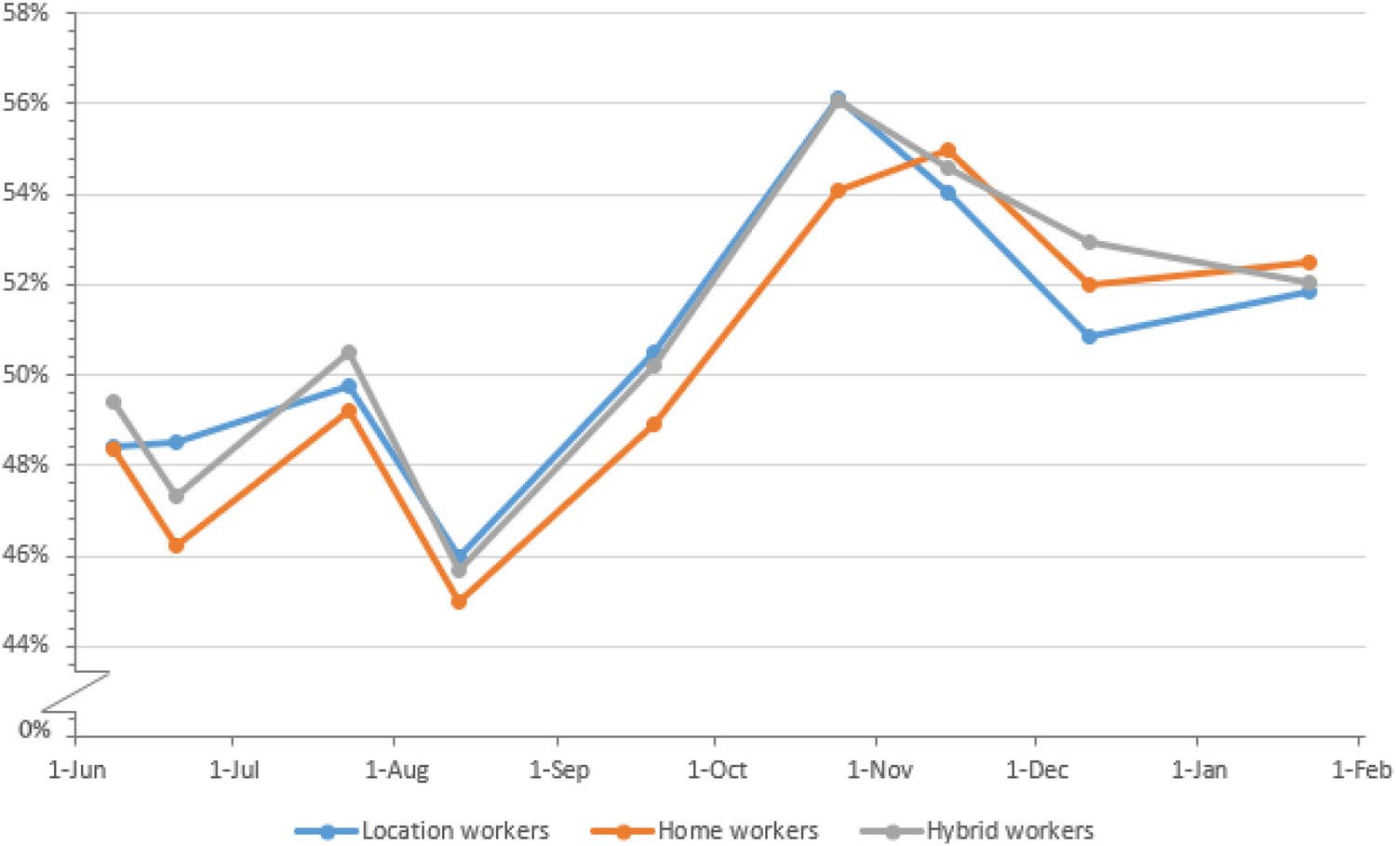
Percentages of participants with some MSP during the pandemic from June 2020 until February 2021, by work situation and date. *N* per round is respectively, 17,211, 16,402, 15,670, 16,537, 16,850, 15,761, 15,786, 14,561, 15,507

Figures 2 and 3 show the percentages of pain in the lower back and total MSP over time from March 2020–February 2021 by work situation. The presence of MSP followed a pattern with somewhat lower occurrences in May and August and a slight increase from August onwards to November. This pattern over time was very similar for each work situation. The presence of pain in the upper back and pain in the neck, shoulder(s) and/or arm(s) over time are shown in Figs. 5 and 6 in Appendix B. Similarly, the presence of upper back pain as well as the presence of pain in the neck, shoulder(s) and/or arm(s) showed a slight increase from August onwards to November without large differences between working situations.

**Fig. 4:**
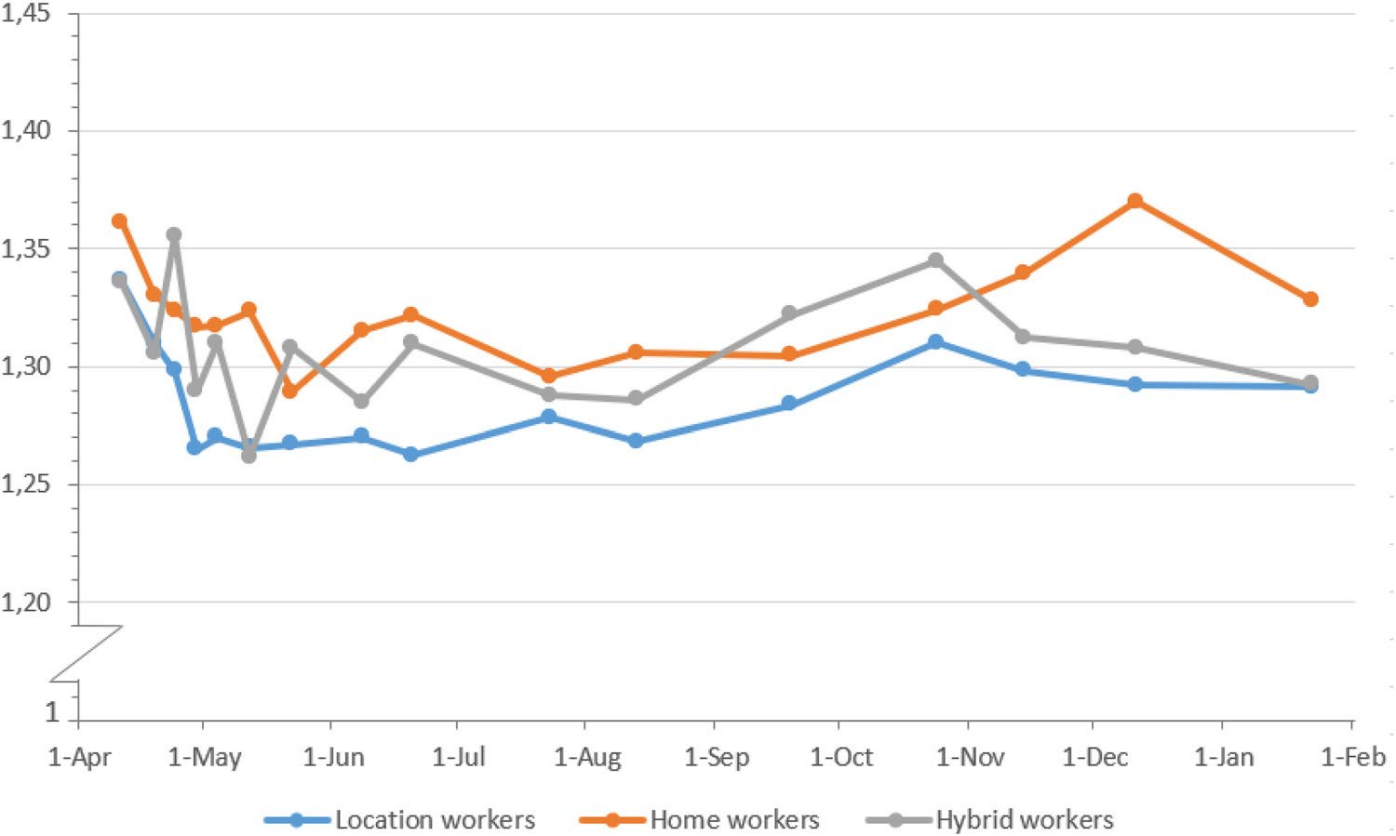
Severity of pain in the lower back during the pandemic from April 2020 until February 2021 among workers reporting any pain, by work situation and date. *N* per round is, respectively, 9645, 9399, 8708, 8386, 7640, 7029, 7316, 6046, 5632, 5655, 5464, 6166, 6312, 6041, 5272, 5693

**Fig. 5:**
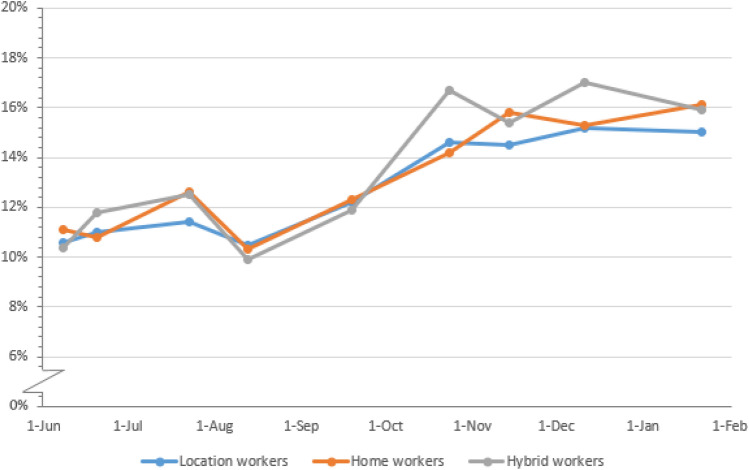
Percentages of participants with pain in the upper back during the pandemic from June 2020 until February 2021, by work situation and date. *N* per round is respectively: 17,223, 16,409, 15,677, 16,547, 16,861, 15,770, 15,799, 14,574, 15,515

**Fig. 6:**
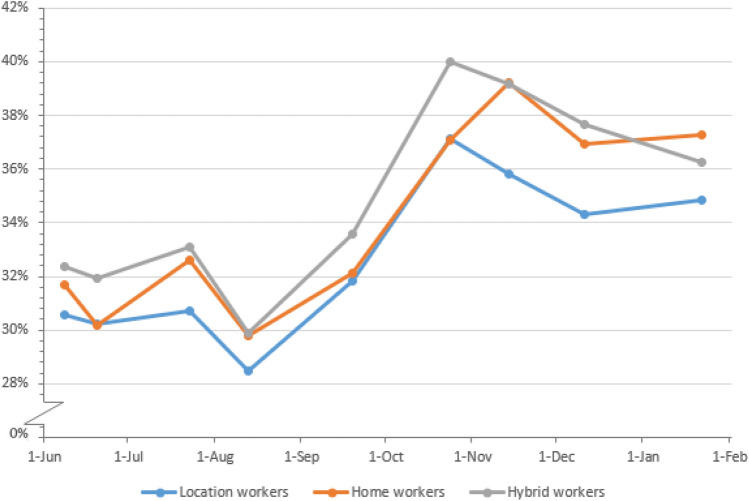
Percentages of participants with pain in the neck, shoulder(s) and/or arm(s) during the pandemic from June 2020 until February 2021, by work situation and date. *N* per round is respectively: 17,228, 16,421, 15,686, 16,552, 16,870, 15,771, 15,800, 14,575, 15,527

**Fig. 7:**
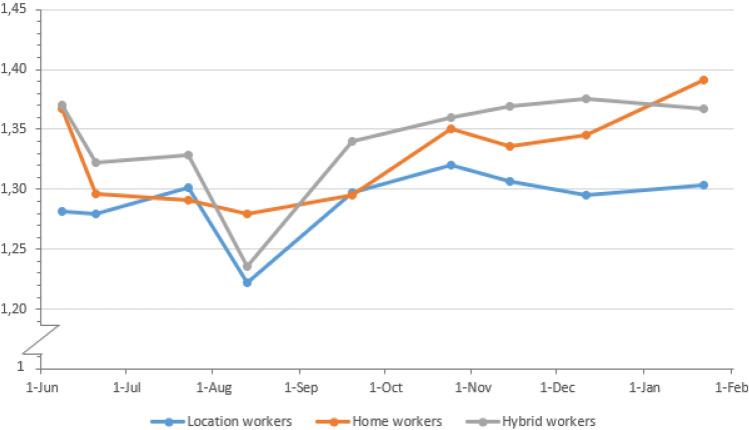
Severity of pain in the upper back during the pandemic from June 2020 until February 2021 among workers reporting any pain, by work situation and date. *N* per round is respectively: 1846, 1808, 1853, 1722, 2060, 2307, 2354, 2243, 2398

**Fig. 8:**
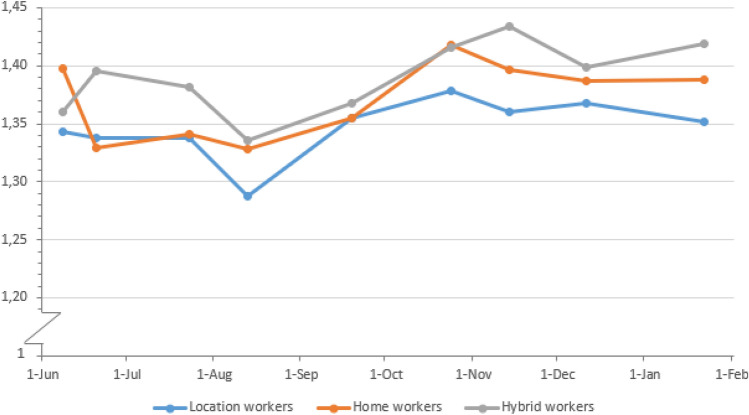
Severity of pain in the neck, shoulder(s) and/or arm(s) during the pandemic from June 2020 until February 2021 among workers reporting any pain, by work situation and date. *N* per round is respectively: 5349, 4982, 4924, 4789, 5406, 5889, 5855, 5145, 5557

Figure 4 shows the severity of the pain in the lower back over time among location, home and hybrid workers reporting any pain (values of zero excluded). From the figure, it can be visually observed that the severity of pain in the lower back was quite stable over time and was quite similar for the three work situation groups, with a mean severity of 1.30 (on scale 1–4) over time. The mean severity of pain over time was 1.30 for those with upper back pain (Fig. 7 in Appendix B) and 1.36 for those with pain in the neck, shoulder(s) and/or arm(s) (Fig. 8 in Appendix B).

### Association between working from home and MSP

The GEE analyses showed that workers who worked from home had an increased risk of pain in all body areas compared to those working on location (Table 2). Working from home was associated with a higher risk of lower back pain [odds ratio (OR) = 1.05, 95% confidence interval (95% CI) 1.02–1.08], upper back pain (OR = 1.24, 95% CI 1.18–1.31) and pain in the neck, shoulder(s) and/or arm(s) (OR = 1.18, 95% CI 1.13–1.22) compared to working on location. For overall MSP, working from home was associated with a higher risk compared to working on location (OR = 1.12, 95% CI 1.08–1.17). In addition, workers who worked from home had an increased risk of relatively more severe pain in all body areas compared to workers who worked on location with ORs ranging from 1.18 to 1.31.

**Table 2:** Results of the GEE analyses on the associations between working situation and presence of MSP and presence of severe MSP, the reference group is location workers

	Presence of MSP (cut-off point: a little bit pain)			Presence of severe MSP (cut-off point: quite a lot pain)		
	Model 0	Model 1	Model 2	Model 0	Model 1	Model 2
	OR (95% CI)	OR (95% CI)	OR (95% CI)	OR (95% CI)	OR (95% CI)	OR (95% CI)
Pain in the lower back						
Working from home	0.99 (0.97–1.02)	1.03 (1.00–1.05)*	1.05 (1.02–1.08)*	1.12 (1.04–1.20)*	1.19 (1.10–1.28)*	1.18 (1.09–1.28)*
Hybrid working	1.00 (0.96–1.03)	1.02 (0.99–1.05)	1.03 (1.00–1.07)	1.09 (0.98–1.20)	1.12 (1.01–1.25)*	1.12 (1.01–1.24)*
Pain in the upper back						
Working from home	1.14 (1.09–1.20)*	1.22 (1.16–1.28)*	1.24 (1.18–1.31)*	1.22 (1.03–1.44)*	1.32 (1.10–1.57)*	1.31 (1.09–1.58)*
Hybrid working	1.04 (0.98–1.11)	1.08 (1.01–1.15)*	1.09 (1.02–1.17)*	1.31 (1.06–1.64)*	1.33 (1.07–1.66)*	1.32 (1.05–1.65)*
Pain in the neck, shoulder(s) and/or arm(s)						
Working from home	1.11 (1.07–1.15)*	1.17 (1.13–1.21)*	1.18 (1.13–1.22)*	1.14 (1.03–1.26)*	1.21 (1.09–1.34)*	1.18 (1.06–1.32)*
Hybrid working	1.01 (1.05–1.15)*	1.13 (1.08–1.19)*	1.14 (1.09–1.20)*	1.17 (1.02–1.33)*	1.19 (1.04–1.36)*	1.17 (1.02–1.34)*
Total MSP						
Working from home	1.05 (1.02–1.09)*	1.10 (1.06–1.14)*	1.12 (1.08–1.17)*			
Hybrid working	1.04 (1.00–1.09)*	1.07 (1.03–1.12)*	1.09 (1.04–1.14)*			

Model 0 is the crude model without covariates, model 1 is adjusted for demographic covariates, model 2 is adjusted for demographic covariates, work covariates and BMI. Reference group: location workers. *N* = 40,702 in the analyses on pain in the lower back and *N *= 28,915 in the analyses on pain in the upper back pain and pain in the neck/shoulder(s) and/or arm(s)

*p<0.05

Except for pain in the lower back, hybrid workers also appeared to have an increased risk of MSP compared to location workers, with ORs varying from 1.09 to 1.14. In addition, workers who worked hybrid during the COVID-19 pandemic had an increased risk of severe pain in all body areas compared to those working on location (ORs varying from 1.12 to 1.32).

### Sensitivity analyses on the association between working from home and MSP

First, including general health in the models confirmed the findings that workers who worked from home had an increased risk of pain in all body areas compared to those working on location during the COVID-19 pandemic with ORs varying from 1.04 to 1.22 (Table 3). The effect estimators on severe MSP became slightly smaller when controlling for general health, with only a significant association between working from home and severe pain in the lower back (OR = 1.13, 95% CI 1.04–1.23). Second, the extra sensitivity analysis on the presence of pain and presence of severe pain within the group workers in business economics and administrative occupations generally confirmed the results of the original analyses as well (Appendix C, Table 6). Third, the analyses in which continuous working from home was compared to hybrid working during the overall study period showed that workers who worked from home continuously were somewhat more likely to have MSP (OR = 1.06, 95% CI 1.02–1.11 for pain in the lower back, OR = 1.11, 95% CI 1.03–1.20 for pain in the upper back and OR = 1.09, 95% CI 1.03–1.15 for pain in the neck/shoulder(s) and/or arm(s)) and severe MSP in the lower back (OR = 1.15, 95% CI 1.03–1.29) (Appendix D, Table 7).

**Table 3:** Results of the sensitivity analyses based on a population of workers who reported their general health (*N* = 33,411) and with general health included in the model, the reference group is location workers

	Presence of MSP (cut-off point: a little bit pain)		Presence of severe MSP (cut-off point: quite a lot pain)	
	Model 1	Model 2	Model 1	Model 2
	OR (95% CI)	OR (95% CI)	OR (95% CI)	OR (95% CI)
Pain in the lower back				
Working from home	1.05 (1.02–1.08)*	1.04 (1.01–1.07)*	1.16 (1.06–1.26)*	1.13 (1.04–1.23)*
Hybrid working	1.03 (0.99–1.07)	1.02 (0.99–1.06)	1.10 (0.98–1.23)	1.08 (0.96–1.21)
Pain in the upper back				
Working from home	1.23 (1.17–1.31)*	1.22 (1.15–1.29)*	1.27 (1.04–1.54)*	1.21 (0.99–1.46)
Hybrid working	1.08 (1.01–1.16)*	1.07 (1.00–1.15)	1.24 (0.98–1.57)	1.20 (0.95–1.52)
Pain in the neck, shoulder(s) and/or arm(s)				
Working from home	1.17 (1.12–1.22)*	1.16 (1.11–1.21)*	1.15 (1.02–1.29)*	1.11 (0.99–1.25)
Hybrid working	1.13 (1.07–1.18)*	1.12 (1.07–1.18)*	1.12 (0.97–1.29)	1.09 (0.95–1.27)
Total MSP				
Working from home	1.12 (1.08–1.16)*	1.11 (1.07–1.16)*		
Hybrid working	1.09 (1.04–1.14)*	1.08 (1.03–1.13)*		

The study populations used are *N *= 33,405 for the analyses on pain in the lower back and *N* = 24,262 for the analyses on the other types of pain. Model 1 is adjusted for demographic covariates, work covariates and BMI. Model 2 is adjusted for demographic covariates, work covariates, BMI and general health. Reference group: location workers

*p<0.05

### Discussion

The aim of the study was to investigate the longitudinal association between working from home during the COVID-19 pandemic and musculoskeletal pain. We studied musculoskeletal pain symptoms over a year period and found that, first, the trends of MSP over time were very similar for each working situation. Second, the results indicate that workers who worked from home during the COVID-19 pandemic had higher risks of having MSP in all body areas compared to workers who worked on location. For hybrid workers, there was some evidence towards having higher risks of MSP in the upper back and MSP in the neck, shoulder(s) and/or arm(s) in this period. Third, the results indicated that both home and hybrid workers have higher risks of relatively severe MSP in different body areas.

The results are in line with a previous study in Turkey which also found an association between working from home and MSP when comparing home workers with location workers (Toprak Celenay et al. 2020), but further studies on this subject appeared to be scarce. The effect estimators in this study were slightly higher for the presence of pain in the upper back and pain in the neck, shoulder(s) and/or arm(s) than for pain in the lower back. Other studies found that office workers with a high computer workload suffer particularly from pain in the upper part of the musculoskeletal system, more than pain in the lower back (Cho et al. 2012), and that inadequate workstation conditions (for example an inappropriate chair height or inadequate arm and back rest) mainly are linked to MSP in upper limbs (Rodrigues et al. 2017). 

Controlling for general health did not produce substantial differences in the results, so it is not likely that the association between working from home and the presence of MSP was due to problems with workers’ general health. The odds ratios in the analyses on severe pain did not change in direction. Some odds ratios of severe pain, however, became insignificant when we adjusted for general health. Non-significant relationships may be explained by a smaller study population in this sensitivity analysis and the subsequently wider confidence intervals. The same holds for the second sensitivity analysis within the population of workers in business economics and administrative occupations. As a result, the greater likelihood of severe pain in the neck, shoulder(s) and/or arm(s) for homeworkers was no longer significant within this sensitivity analysis. We also looked at working from home continuously over the study period. The third sensitivity analysis indicated that workers who worked exclusively from home throughout the study period were somewhat more likely to have MSP than those who worked occasionally from home. There is thus some change in presence of MSP as the duration of work from home increases. Here, we made the assumption that workers who reported to work from home during all the questionnaire rounds in which they participated, did not work on location during time when they did not participate.

For future research it would be interesting to investigate whether or not the design of the home workstation is possibly an underlying mechanism which can explain MSP for homeworkers during the COVID-19 period. In the current study there was no information on the design of the home workstation available. In the beginning of the pandemic, workers were unprepared to work from home and the transition from working on location to working from home took place very rapidly because of the urgency of the COVID-19 pandemic. The quick transition to working from home may have resulted in unfavorable workstations and a lack of policies to support healthy home working environments. It is possible that there was too little space in the home for the home worker(s) and that home workers were forced to sit for long times in chairs and behind desks that were ergonomically unsuitable which increased home workers’ MSP. On the other hand, one would expect that improvements to the home office were made during the study period because of the persistent need to work from home (Oude Hengel et al. 2021). In that particular case, MSP would have decreased over time because working conditions at home were improved. However, the trend lines show no remarkable decrease, nor increase, in musculoskeletal pain symptoms over time. Therefore, other mechanisms may as well underlie the association between working from home and MSP. For example, previous studies suggest that home workers had reduced physical activity and prolonged sedentary behavior during the pandemic in comparison with before the pandemic (Fukushima et al. 2021; Loef et al. 2022), which in turn may have contributed to the development of MSP (Kastelic et al. 2018; Lim and Pranata 2021; Søgaard and Sjøgaard 2017). More research is needed to examine the interactions between these factors, and the contributions of these factors to the onset of MSP among home workers.

A limitation of this study is the lack of information about the work situation and MSP before the COVID-19 pandemic. We could not account for the work situation before the COVID-19 pandemic and if workers were already working from home at that time, the work situation before the COVID-19 pandemic may have affected the MSP of those workers during our study period. We also did not have information on baseline MSP measurements prior to the COVID-19 pandemic to see how many and which workers experienced MSP before the COVID-19 pandemic and the change in MSP during the COVID-19 pandemic. The lack of information on MSP before the COVID-19 pandemic may be problematic since previous results suggest that MSP of location and home workers may have declined somewhat during the COVID-19 pandemic (Oude Hengel et al. 2021). In addition, the measures of MSP during the COVID-19 pandemic were self-reported. Because the questions on MSP were about pain in the past 7 days, and later past 14 days, recall bias may exist and may also have increased. Last, we could not account for the number of hours worked, whereas we would expect that the time worked from home per week does matter for MSP. However, a major strength of the current study is its longitudinal design with multiple measurement of work situation and different pain types over almost one year in the COVID-19 pandemic. To our knowledge, the current study is the first study to report a longitudinal association between working from home and MSP.

Knowledge on the association between working from home and MSP during the COVID-19 pandemic may facilitate future policy making with the goal of improving the work situations for many workers who will continue to work from home (at least partly). Policies aimed at reducing MSP are relevant since the prevalence of MSP is associated with sleeping problems, overall fatigue and the mental well-being of workers (Safety et al. 2020). Which prevention measures are suitable does depend on which mechanisms are at play. In case that future research shows that the design of the workstation does matter, prevention measures for employers could for example be diffusion of simple and pragmatic messages on ergonomics and providing financial contributions to equipment (e.g., adjustable chairs) (Bouziri et al. 2020). However, it should also be recognized that appropriate equipment and adaptable furniture alone do not guarantee adequate usage. Workers could for example be trained to optimally set up their furniture or receive technical assistance with the installation (Montreuil and Lippel 2003). In addition, policy-makers could focus on providing simple practical risk assessment tools and guides for workers (Safety et al. 2020). In doing so, it could be of importance that physical activity is promoted and that prolonged sitting is interrupted. For example, the implementation of a software program which encourages workers to take regular breaks can help with the recovery of neck and upper extremity pain (Van den Heuvel et al. 2003). In our study, we adjusted for occupational class. In future studies, it is of importance to take into account job characteristics and psychosocial risk factors more extensively. For example, psychological distress, high workload and little influence over one's own work situation were found as predictors of MSP (Bongers et al. 2006; Eltayeb et al. 2007).

This longitudinal designed study showed that working from home conceivably has negative consequences on the musculoskeletal system. Working from home also has several advantages, for example a reduction in commuting time and improved opportunities to combine work and private life (De Macêdo et al. 2020). Therefore, working from home is expected to become more normal practice for many workers. Future policies should pay attention to the negative effects on workers’ MSP. Furthermore, future studies to the mechanisms are recommended as these can be starting points for measures to prevent MSP of home workers.

## Data Availability

The datasets analyzed during the current study are available from the Lifelines Cohort study (https://www.lifelines.nl/researcher) but restrictions apply to the availability of these data, which were used under license for the current study, and so are not publicly available. Requests to access these data should be directed to Lifelines Research Office (research@lifelines.nl).

## Appendix A

See Tables 4, 5.

**Table 4:** Overview of the 16 questionnaire rounds and number of observations used in the current study from the lifelines COVID-19 cohort from March 2020 to February 2021

Round	Date	Determinant: work situation	Outcomes: pain in the lower back (LB), pain in the upper back (UB), pain in the neck, shoulder(s) and/or arm(s) (ANS)	*N* of included workers per round for outcomes, LB, UB and ANSrespectively
1	Mar to Apr 2020	Work situation	Pain in LB	24,210
2	Apr to May 2020	Work situation	Pain in LB	23,811
3	Apr to May 2020	Work situation	Pain in LB	23,418
4	Apr to May 2020	Work situation	Pain in LB	23,776
5	Apr to May 2020	Work situation	Pain in LB	22,286
6	Apr to May 2020	Work situation	Pain in LB	20,393
7	May 2020	Work situation	Pain in LB	20,661
8	May to Jun 2020	Work situation	Pain in LB + UB + ANS	17,244, 17,223, 17,228
9	Jun 2020	Work situation	Pain in LB + UB + ANS	16,435, 16,409, 16,421
10	Jul 2020	Work situation	–	–
11	Jul to Aug 2020	Work situation	Pain in LB + UB + ANS	15,708, 15,677, 15,686
12	Jul to Sep 2020	Work situation	Pain in LB + UB + ANS	16,564, 16,547, 16,552
13	Sep 2020	Work situation	Pain in LB + UB + ANS	16,888, 16,861, 16,870
14	Oct to Nov 2020	Work situation	Pain in LB + UB + ANS	15,774, 15,770, 15,771
15	Nov 2020	Work situation	Pain in LB + UB + ANS	15,807, 15,799, 15,800
15b	Nov to Dec 2020	–	Pain in LB + UB + ANS	–
16	Dec 2020	Work situation	Pain in LB + UB + ANS	14,575, 14,574, 14,575
16b	Dec 2020 to Jan 2021	–	Pain in LB + UB + ANS	–
17	Jan to Feb 2021	Work situation	Pain in LB + UB + ANS	15,530, 15,515, 15,527

Rounds 10, 15b and 16b from the Lifelines COVID−19 cohort were excluded in the current study due to the questions of the determinant or outcome measures not being included in these survey rounds

**Table 5:** A distribution of the coding across occupational classes according to the occupational classification of Statistic Netherlands (BRC ROA-CBS 2014)

Code	Occupational class
1	Pedagogical occupations
2	Creative and linguistic occupations
3	Commercial occupations
4	Business economics and administrative occupations
5	Managers
6	Public administration, security and legal occupations
7	Technical occupations
8	ICT occupations
9	Agricultural occupations
10	Care and welfare occupations
11	Service occupations
12	Transport and logistics occupations
13	Not elsewhere classified

## Appendix B

See Figs. 5, 6, 7 and 8

## Appendix C

See Table 6.

**Table 6:** Results of the GEE analyses on the associations between working situation and presence on MSP and presence of severe MSP, within workers with business economics and administrative occupations, the reference group is location workers

	Presence of MSP (cut-off point: a little bit pain)			Presence of severe MSP (cut-off point: quite a lot pain)		
	Model 0	Model 1	Model 2	Model 0	Model 1	Model 2
	OR (95% CI)	OR (95% CI)	OR (95% CI)	OR (95% CI)	OR (95% CI)	OR (95% CI)
Pain in the lower back						
Working from home	1.08 (1.02–1.13)*	1.10 (1.04–1.16)*	1.10 (1.04–1.16)*	1.24 (1.07–1.43)*	1.29 (1.10–1.50)*	1.30 (1.11–1.51)*
Hybrid working	1.02 (0.96–1.09)	1.03 (0.97–1.10)	1.03 (0.97–1.10)	1.14 (0.94–1.38)	1.15 (0.95–1.41)	1.16 (0.95–1.41)
Pain in the upper back						
Working from home	1.17 (1.07–1.28)*	1.19 (1.08–1.30)*	1.20 (1.09–1.31)*	1.40 (1.01–1.93)*	1.46 (1.04–2.04)*	1.47 (1.04–2.06)*
Hybrid working	1.08 (0.97–1.21)	1.08 (0.96–1.21)	1.08 (0.97–1.21)	1.46 (1.01–2.11)*	1.42 (0.98–2.07)	1.42 (0.97–2.06)
Pain in the neck, shoulder(s) and/or arm(s)						
Working from home	1.18 (1.10–1.26)*	1.19 (1.11–1.28)*	1.19 (1.11–1.28)*	1.14 (0.96–1.37)	1.17 (0.97–1.41)	1.16 (0.96–1.40)
Hybrid working	1.15 (1.06–1.25)*	1.15 (1.06–1.25)*	1.15 (1.06–1.25)*	1.16 (0.92–1.45)	1.14 (0.90–1.43)	1.13 (0.90–1.48)
Total MSP						
Working from home	1.16 (1.09–1.23)*	1.18 (1.10–1.26)*	1.18 (1.10–1.26)*			
Hybrid working	1.11 (1.02–1.20)*	1.12 (1.03–1.21)*	1.12 (1.03–1.21)*			

The study populations are *N*=9557 for the analyses on pain in the lower back and *N*=6857 for the analyses on the other types of pain. Model 0 is the crude model without covariates, model 1 is adjusted for demographic covariates, model 2 is adjusted for demographic covariates, work covariates and BMI. Reference group: location workers

**p*<0.05

## Appendix D

See Table 7

**Table 7:** Results of the GEE analyses on the associations between the overall home working over the complete study period and presence of MSP and presence of severe MSP, the reference group is hybrid workers (throughout the study period)

	Presence of MSP (cut-off point: a little bit pain)			Presence of severe MSP (cut-off point: quite a lot pain)		
	Model 0	Model 1	Model 2	Model 0	Model 1	Model 2
ORs working from home	OR (95% CI)	OR (95% CI)	OR (95% CI)	OR (95% CI)	OR (95% CI)	OR (95% CI)
Pain in the lower back	1.05 (1.00–1.10)*	1.05 (1.00–1.10)*	1.06 (1.02–1.11)*	1.17 (1.04–1.30)*	1.16 (1.04–1.29)*	1.15 (1.03–1.29)*
Pain in the upper back	1.05 (0.98–1.14)*	1.09 (1.01–1.17)*	1.11 (1.03–1.20)*	1.05 (0.84–1.30)	1.13 (0.91–1.40)	1.18 (0.94–1.48)
Pain in the neck, shoulder(s) and/or arm(s)	1.05 (1.00–1.11)	1.09 (1.03–1.15)*	1.09 (1.03–1.15)*	1.04 (0.91–1.18)	1.09 (0.95–1.25)	1.08 (0.94–1.25)

Model 0 is the crude model without covariates, model 1 is adjusted for demographic covariates, model 2 is adjusted for demographic covariates, work covariates and BMI. Reference group: hybrid workers

**p*<0.05

## Declarations

### Protection of research participants

Approval of the Lifelines Cohort study was obtained from the Medical Ethics Committee of the University Medical Center Groningen, The Netherlands. Informed consent was obtained from all participants.
